# An Optical Algorithm for Relative Thickness of Each Monochrome Component in Multilayer Transparent Mixed Films

**DOI:** 10.3390/polym14163423

**Published:** 2022-08-22

**Authors:** Meiqin Wu, Zuoxiang Lu, Yongrui Li, Xiaofei Yan, Xuefei Chen, Fangmeng Zeng, Chengyan Zhu

**Affiliations:** 1 College of Textile Science and Engineering (International Institute of Silk), Zhejiang Sci-Tech University, Hangzhou 310018, China; 2 Key Laboratory of Intelligent Textile and Flexible Interconnection of Zhejiang Province, Zhejiang Sci-Tech University, Hangzhou 310018, China

**Keywords:** film thickness, image, transmission, scattered photometric, Kubelka-Munk

## Abstract

A modification of the two-flux Kubelka-Munk (K-M) model was proposed to describe the energy conservation of scattered light in colored mixed material with a defined scattered photometric, which is applied for the relative quantity distribution of each colored monochrome component in mixed material. A series of systematical experiments demonstrated a higher consistency with the reference quantity distribution than the common Lambert-Beer (L-B) law. Its application in the fibrogram of each component for measuring the cotton fiber’s length was demonstrated to be good, extending its applicability to white and dark colored blended fibers, the length of which is harder to measure using L-B law.

## 1. Introduction

The quantity or relative quantity distribution of each monochrome item is an important variable to configure a color-mixed material’s properties in terms of structure and uniformity in the textile industry, such as the color blending fibers, yarn, and fabric. A potential environmental textile brand featured blending fibers after fiber coloring, with 50% reduction of water than traditional process [1,2,3]. Meanwhile, it is proposed to be applied in the field of photometric measurement, among which the fibrogram is a typical application, a way for measuring the length of white cotton fiber by the parallel fiber beard linear density along the fiber axis [4,5].

In 1932, Hertel [4,5] proposed a modified form of the derived Lambert-Beer (L-B) law to measure the linear density of cotton beards for bias reduction. However, experimental coefficients were different for various materials and obtained difficultly, calling for theoretical study [4,5,6,7,8,9]. In 1970s, the high volume instrument (HVI) was invented by Spinlab Corporation based on Hertle’s study for cotton fiber length distribution, and has been an international standard method on white cotton fiber length until now [9]. In 2016, Wu et al. [10] derived a modification of two-flux Kubelka-Munk (K-M) theory for relative thickness or surface density of a turbid medium. The theory contains the up- and down-wards absorption and scattering light overcoming the L-B law’s shortcoming of including down-wards absorption only [10,11,12]. Its results proved to be much better than L-B law, particularly for scattered wool fibers [10,11,12]. Although the derived transmittance K-M theory has been widely used in predicting the relative thickness of white materials [10,11,12], the mixed-colored specimen of each monochrome fibers has not yet been investigated which is typical important for quality control of color blending yarn industry.

In 2021, Chen et al. [13,14] of our group proposed an optical algorithm for the thickness of each color material in a mixed multilayer transparent specimen, combined L-B law and transmission images. An estimating equation group was developed to describe the relationship between the physical thickness of each color material and the optical depth of multilayer transparent specimen under different monochrome light from linear regression methods. The binary system of first order equations was employed to predict each colored wool fiber material’s relative physical thickness in the mixed colored-fiber specimen. Although its results turn to be pretty good in smooth films, the L-B law’s shortcoming of containing down-wards absorption only limits its usage scope in scattering and thick films [13] and fiber materials [14], particularly the fiber beards with different colors [14]. Hence, relative quantity distribution of each monochrome component needs further study.

In this paper, combined with the previous derived transmission K-M theory and conservation law of light flux of scattered light, a new scattering optical algorithm is proposed for the relative thickness of each color material in a multilayer transparent specimen. This algorithm has an advantage of comprising up- and down-ward scattering and absorption lights, overcoming L-B law’s shortcomings of including down-ward absorption light only. In this optical algorithm, the linear regression method was applied in obtaining linear equation between the physical thickness and optical scattered photometric of multilayer transparent monochrome specimens. According to the conservation law of light flux of scattered light, ab estimation equation system was expressed to predict relative thickness of each colored material in the multilayer specimen, and a better affinity is achieved according to the comparison between experimental and predicted relative thickness which is compared with results of previous algorithm from L-B law.

## 2. Theory

### 2.1. Lambert-Beer Law

L-B law [8,9,10,11,12,13,14,15] provides a light absorptivity relationship between the attenuation of light and the physical thickness of material when a light transmits through a material. This relationship only considers the down-wards light absorption, expressed as in Equations (1) and (2),

(1) $$I=I_{0}e^{-Kx}$$

(2) $$A=xK=\ln(\frac{I_{0}}{I})=-\ln(T)$$

where *K* is the absorption coefficient, *x* is the thickness of the material, *T* is the transmittance, and *A* is the absorbance.

### 2.2. Derived Kubelka-Munk Theory

Considering the up- and down-wards light scattering and absorption, Wu et al. [12], the author, published a derived K-M theory indicating a scattering relationship between the transmittance of light and the physical thickness of material when light transmits through a material. According to the equation, the thickness (*x*) of specimen is proportional to the algorithm of transmittance as elaborated in Equation (3),

(3) $$P=Sx=\frac{2r_{\infty }}{1-r_{\infty }^{2}}\ln(\frac{1-r_{\infty }^{2}+\sqrt{{\left(1-r_{\infty }^{2}\right)}^{2}+4T^{2}r_{\infty }^{2}}}{2T})$$

where, *S* denotes the coefficient of scatter defined by the corresponding thickness of layer; $r_{\infty }$ is the light reflectivity of the material with infinite thickness; *P* is defined as scattered photometric here representing the ability of material’s light scattered. Here, *S* could be obtained experimentally.

According to the conservation law of light flux of scattered light, the scattered coefficients of color-mixed material is equal to the sum of the results of scattered coefficient of each composition multiplied by its corresponding concentration ratio $w_{i}$, *S*_mix_ =$\;\sum_{i=1}^{n}w_{i}S_{i}$, where *w*_i_ = *x*_i_/*x*_mix_ and *n* is number of the monochrome materials. 

Hence, the scattered photometrics of mixed material is proposed in this paper to be equal to the sum of the scattered photometrics of its corresponding monochrome materials, expressed as *P*_mix_ = $\sum_{i=1}^{n}P_{i}$. Two or three of these equations under different lights form the mixed-film estimation equation system for 2-mixed or 3-mixed color multilayer films, respectively.

### 2.3. Proposed Estimation Procedure

In this study, our own built imaging scanner was applied to obtain the RGB transmission images at a greyscale of 0–255 with a resolution of 1000 dpi, where dpi means the number of points within per inch. These acquired R, G, and B values represent the transmitted light of red (R), green (G), and blue (B) monochromatic light, respectively. 

**Figure 1:**
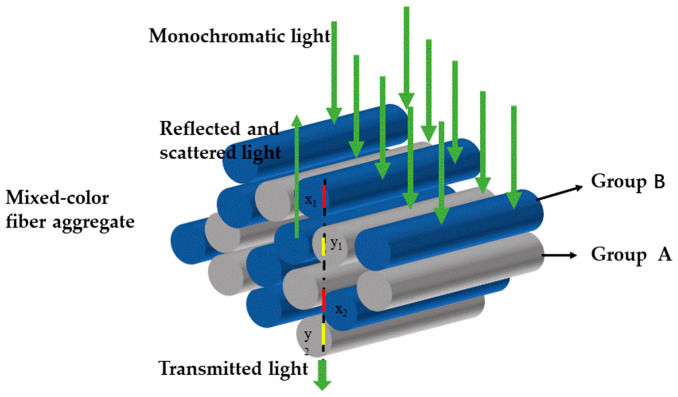
Monochromatic light incident analysis of two-color hybrid fiber aggregates.

Figure 1 shows that when monochromatic light enters the fiber aggregate, it is assumed that both light reflection and light scattering inside the fiber aggregate are considered as scattering, while scattering and absorption in air are ignored. According to the above conservation law of scattered light flux, for A and B two-color mixed color fiber, the scattered light amount of the mixed color fiber is equal to the total scattered light of component A and the total scattered light of component B.

**Figure 2:**
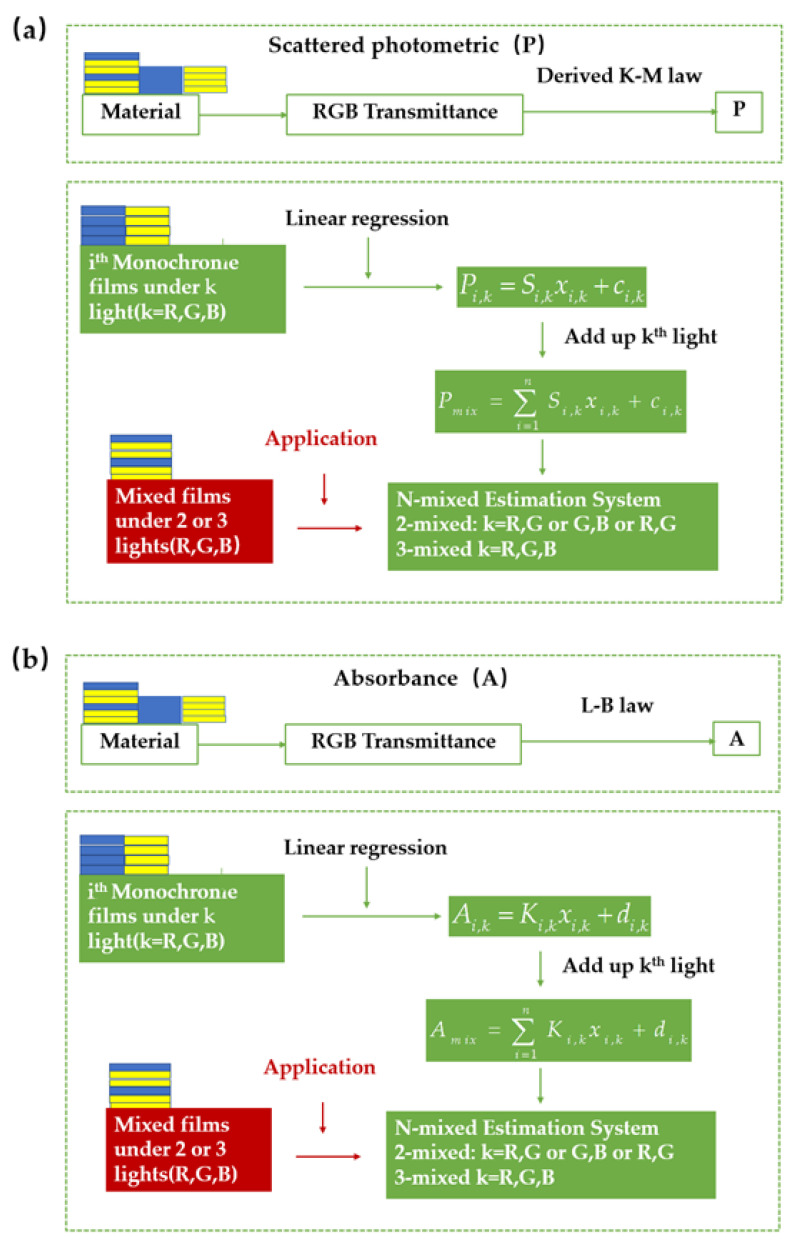
Flow chart to achieve relative thickness of each color film in the multilayer films, (**a**) Scattered photometric (P), (**b**) Absorbance (A).

Figure 2 shows the flow chart to achieve the physical thickness of each color film in the multilayer specimen, in which ith (*i* = 1, 2, 3) film represents different monochromatic film and kth (*k* = R, G, B) light denotes the light channel of color images. This procedure has two steps: (1) Color-mixed estimation system and (2) Application. For color-mixed estimation system, the monochrome films were piled up to multilayer films one by one to scan their transmitted RGB digital images. Next, calculate their corresponding transmittance using R/R_0_, G/G_0_ and B/B_0_, where R_0_, G_0_ an B_0_ represents the amount of incident light under each channel. These transmittance and corresponding infinite reflectance were applied in Equations (1) and (2) to get $A_{i,k}$ and $P_{i,k}$, respectively. Details of reflectance measurement are described in Section 3.2 Optical parameter. After that, train the estimating equations referred to photometric with linear regression method of ith monochromatic films and each light. Furthermore, these estimating equations were added up to form the mixed-film estimation equation under each light. Two or three of these equations under different lights form the mixed-film estimation system for 2-mixed or 3-mixed color multilayer films, respectively. In step 2: Application, the designed mixed films were arranged according to their corresponding designed order and number of a group and accumulated to multi-groups. Next, the RGB images of these groups were scanned to get their transmittance using R/R_0_, G/G_0_ and B/B_0_. These transmittance and corresponding infinite reflectance were applied to obtain the absorbed and scattered photometric, $A_{mix,k}$ and $P_{mix,k}$, using Equations (1) and (2), respectively. Afterwards, these results are substituted into the mixed-film estimation equation system above. 

## 3. Experiment

### 3.1. Material

In this experiment, seven commercial transparent and uniform films with different colors are chosen as the experimental materials, numbered 1# to 7#, whose information are listed in Table 1. Film 1# to 5# are made of poly-ethylene terephthalate (PET) and film 6# to 7# are polypropylene (PP) films. All these colored films have characteristics of transparent and smoothy, except 6# and 7# with rough surfaces. All images in Table 1 were captured from films with 20 layers except 5# containing 40 layers for higher transparency. These samples were employed to build the estimation equation systems for color separation.

To test the proposed method, 8 sets of mixed multilayer films, numbered a# to h# were designed in accordance to their corresponding order and ratio given in Table 2. For example, a group of a# mixed films turns to be 211 arranged from bottom to top, where 2 and 1 stands for a layer of 2# and 1# film respectively. Different numbers of groups are selected for the limit linear test range. Films a#–f# and film g# are of PET and PP respectively, while h# is a mixture of PP and PET with rough and smooth surfaces. a#–h# samples in Table 2 are divided into 5 sets by the compositions of each mixed material, numbered A# to E#. An estimation linear equation system of each set could be composed for thickness or quantity of monochrome material.

### 3.2. Optical Parameter $r_{\infty }$

Reflectance of infinite layers $r_{\infty }$, is an essential optical parameter for derived K-M theory. To measure this parameter, specimens need to be piled up to enough thickness, so that no light can transmit. Samples of monochrome and mixed films are stacked to 20 layers and 10 groups of layers respectively, except 5# with 40 layers for higher transparency. Table 3 denotes the reflectance of infinite layers from reflective images of these multilayers obtained with built scanning image equipment.

### 3.3. Monochrome Estimation Equation

**Figure 3:**
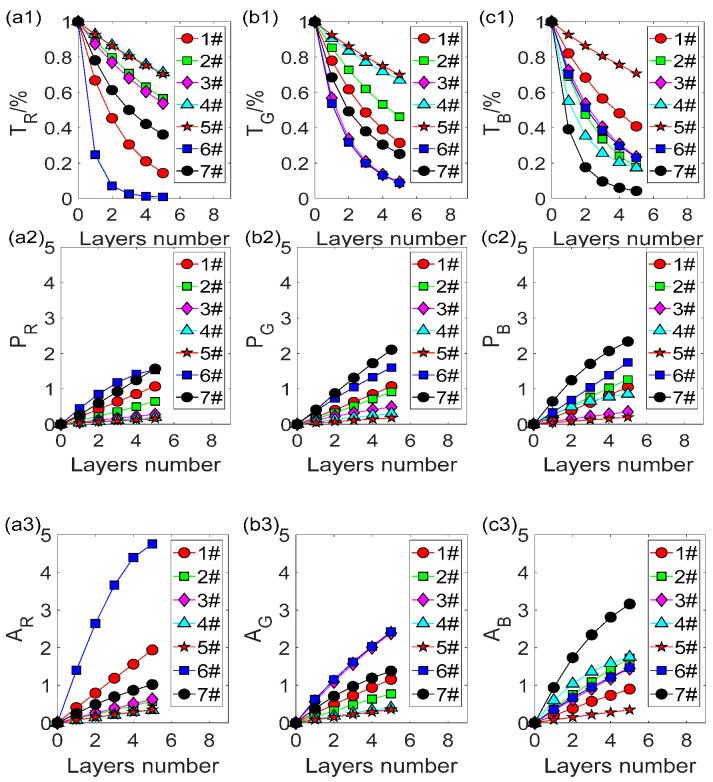
Relationship between transmittance (**a1**–**c1**), scattered photometric (**a2**–**c2**) and absorbance (**a3**–**c3**) of 0–5 layers with 1#–7# films under R, G and B lights.

To get the linear equation between the scattered photometric and thickness, specimens with same color were accumulated to multilayers for RGB images of 1# to 7# samples using a scanner, as shown in Figure 1. Scan images of each colored film with multilayers at an area of 9 mm × 10 mm, ranging from 0–5 layer. Afterwards, their scattered and absorbance were computed with Equation (2) and Equation (1), respectively. Figure 3 indicates the transmitted intensity, absorbed and scattered photometric of 0–5 layers with sample 1# to 7# under R, G and B lights. As physical thicknesses are multiples of its layer numbers, the latter was used as reference values here. It can be seen that the scattered photometric has a better linearity than the absorbed photometric with the layer of films for most films ranging from 0 to 5 layers, such as 7# in R and G channels, and different colored film shows different linearities in R, G and B channels. Hence, optimal channels could be selected according to the transmittance under R, G, and B channels of the 2-mixed mixture and its components. 

In addition, linear regression method was employed for linear equation between the layer number and absorbance or scattered photometric. Data of 0–4 layers for 1#–5# and 0–3 layers for 6#–7# were regressed for better linearity of scattered photometric P = SX + C, and absorbed photometric A = KX + D, where S denotes the coefficient of scatter defined by the corresponding thickness of layer; and K is the coefficient of absorption, and C and D are constants related to noises. In this section, the experiments results indicated good linear relationship between photometric and layer numbers of monochrome films under each monochrome light, whose r^2^ were all above 0.98. These estimated scattering and absorption linear equations could be used in the construction of color-mixed equations complied with the conversation of scattered and absorbed light in the following section. 

### 3.4. Thickness of Each Component in Mixed Samples

**Figure 4:**
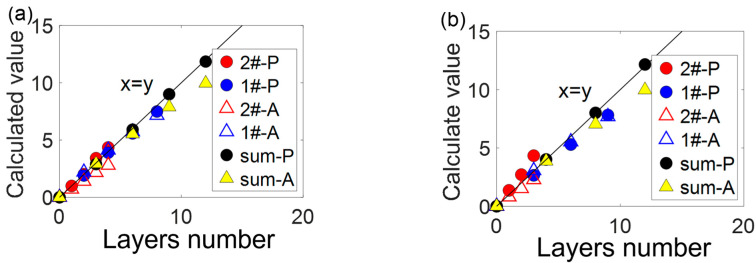
Comparison between physical and calculated monochrome film layers number, in mixed samples of (**a**) Sample a# and (**b**) Sample b# using scattered photometric and absorbance.

To examine the accuracy of proposed method, specimens a# and b# were created numbered A# set as listed in Table 2. Following the conversation of scattered and absorbed light in the following section, estimation equation system was constructed by summing up linear equations from Figure 3 for each component, 1# and 2#, in mixed samples of A# about scattered photometric and absorbance under particular (G or B) light, respectively. Its results comprised 2-mixed estimation equation systems of A#. Figure 3 declared a higher degree of linearity between predicted and measured scattered photometric compared with that of the absorbance from L-B law. This may lead to more accurate predicted layer numbers for scattered method. Afterwards, compute layer number of colored films 1# and 2# with 2-mixed estimation systems of Figure 2. Their results and the sum of each component with both methods are illustrated in Figure 4.

For accuracy analysis, their corresponding layer number deviation ratios σ of each composition were calculated according to Equation (4),

(4) $$\sigma =\frac{\left|x-x_{0}\right|}{x_{0}}\times 100\%$$

where *x* denotes the calculated value; $x_{0}$ is the reference value.

The mean and maximum value of these deviation ratios turns to be 2.05%, 6.21% and 4.14%, 20.40% for derived K-M and L-B methods, respectively. Particularly, the sum of each component measured with the derived K-M has presents less difference from the true data. Hence, the scattered method exhibits a better consequence in a# and b# PET materials compared with L-B law. 

### 3.5. Relative Thickness of Each Component in Mixed Material

**Figure 5:**
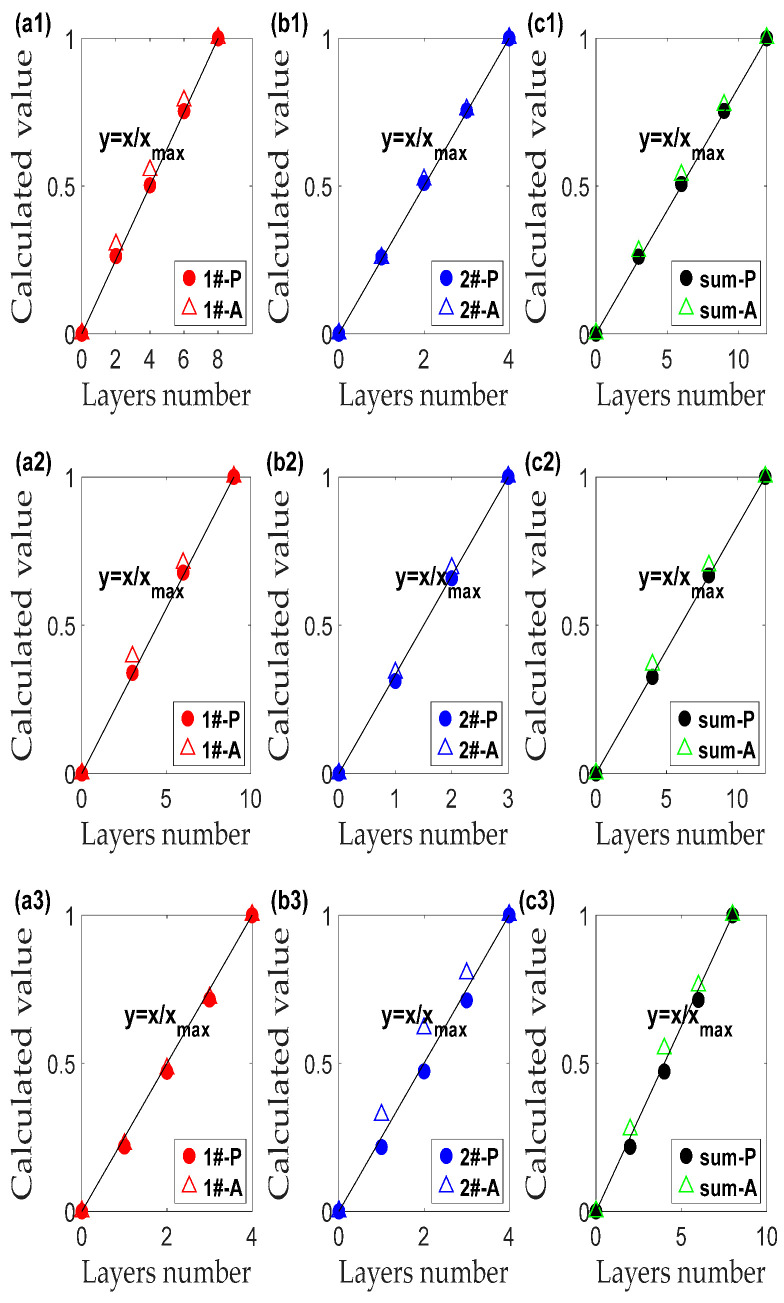

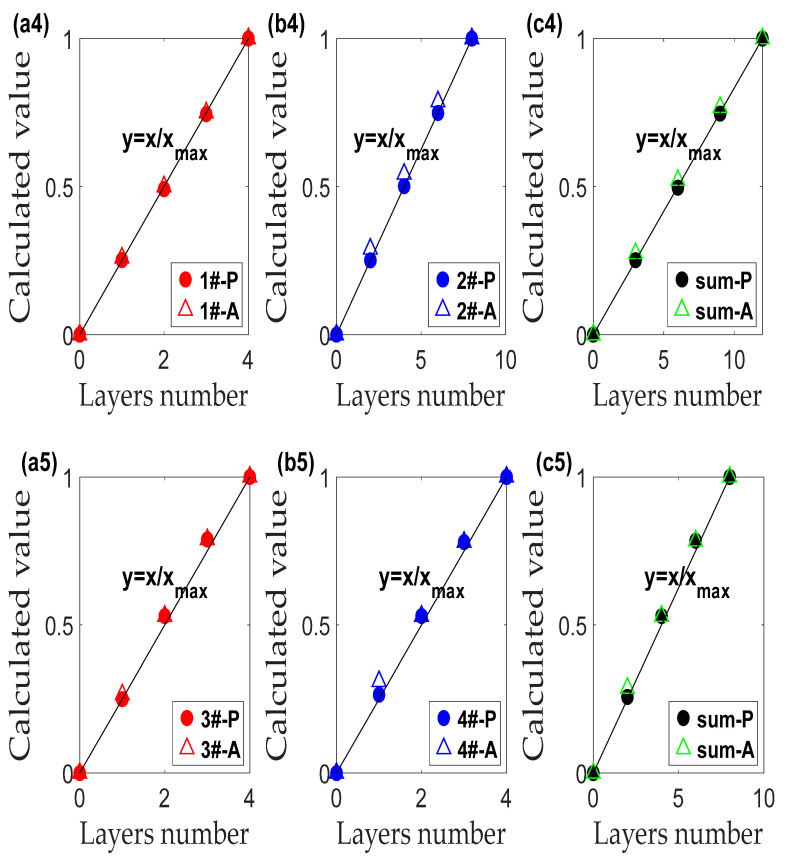

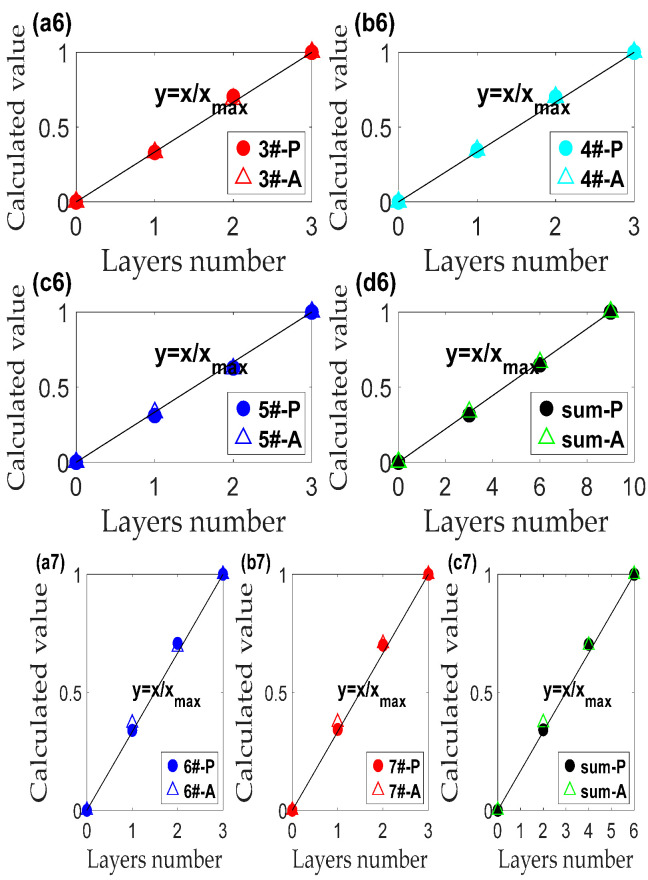

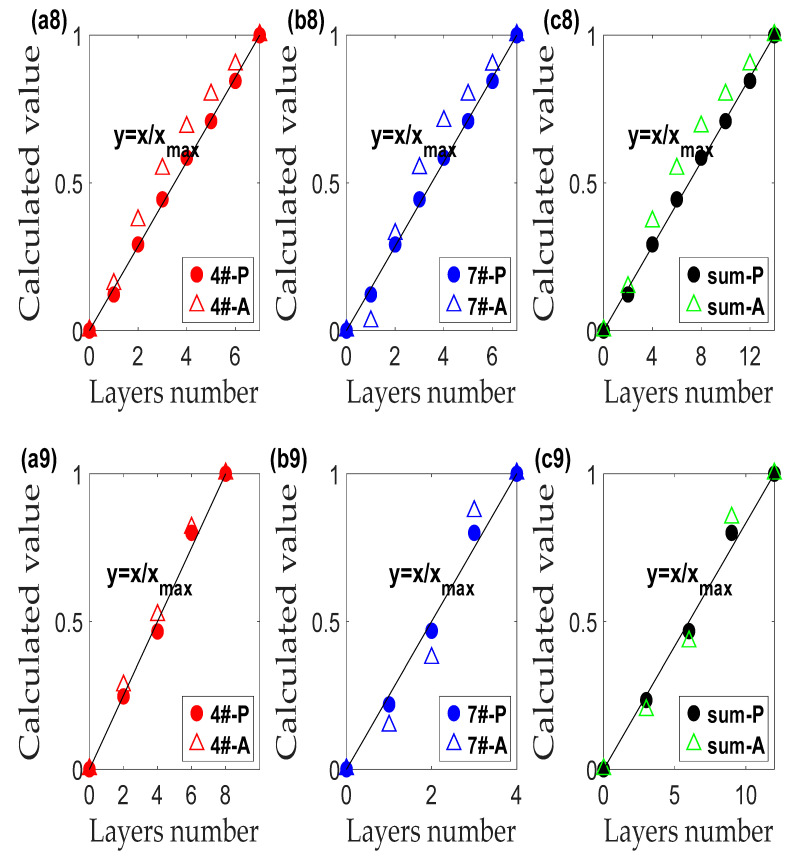
Comparison between layers number and calculated relative thickness (calculated value) of each component and their sum for Sample a# (**a1**–**c1**), b# (**a2**–**c2**), c# (**a3**–**c3**), d# (**a4**–**c4**), e# (**a5**–**c5**), and f# (**a6**–**d6**) with PET material, g# (**a7**–**c7**) with PP materials and h# (**a8**–**c8**), and i# (**a9**–**c9**) with PP\PET mixed material.

To avoid effect of boundary reflectance ignorance, random error of reflectance of infinite layers and noises of equipment [8,16], relative optical thickness was proposed for testing the quantity distribution of each monochrome material. PET, PP and PP\PET mixed materials were applied for relative optical thicknesses of monochromatic film in multilayers as Table 2 described. For estimation system construction, add up the linear equations of each component from 0–4 layers of colored films in Figure 3, following procedures in Figure 2 to a predicted mixed equation under a particular light (R, G and B). Afterward, two or three of these predicted equations under different monochromatic light were employed to construct the 2- or 3-mixed estimation equation system. Finally, the relative thickness for each composition of the mixed multilayers was calculated with Equation (5), shown in Figure 5.

(5) $$x_{r}=\frac{x-x_{\min}}{x_{\max}-x_{\min}}\times 100\%$$

Relative thickness deviation ratios of Figure 5 were computed with Equation (4) as shown in Table 4, whose mean and maximum values from scattered photometric and absorbance were 3.56%, 14.03% and 6.77%, 42.24%, for 2-mixed PET material (1#2# and 2#1#), 2.28%, 6.86% and 1.7%, 6.74% for 3-mixed PET material (4#5#3#), 1.94%, 6.13% and 4.08%, 12.05% for 2-mixed PP material (6#7#), and 2.89%, 14.38% and 17.07%, 78.31% for PET/PP material (7#4#), respectively. This indicates a better application of the modification of K-M theory than L-B law both in average and maximum error rate. The reason for this is that the L-B model only considers the unidirectional absorption of light by materials but does not consider the reflection and scattering of light. When the light passes through the material, in addition to absorption and reflection, a large amount of scattered light will be generated inside and on the surface. Therefore, the modified K-M theory is proposed to consider not only light absorption and light transmission, but also light scattering. Its advantages are particularly obvious in PP\PET mixed sample with smooth and rough surfaces.

## 4. Application

**Figure 6:**
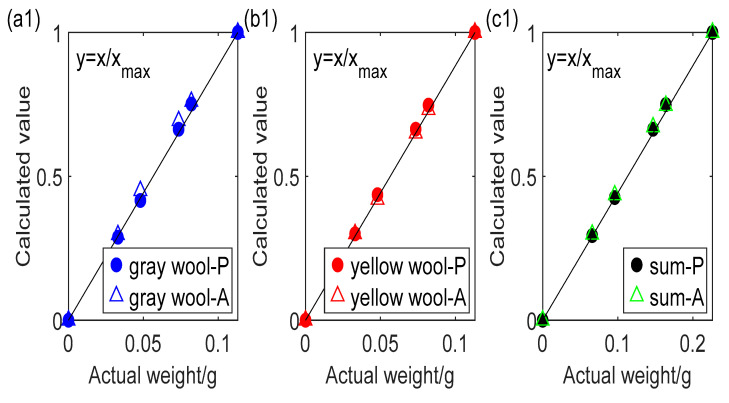

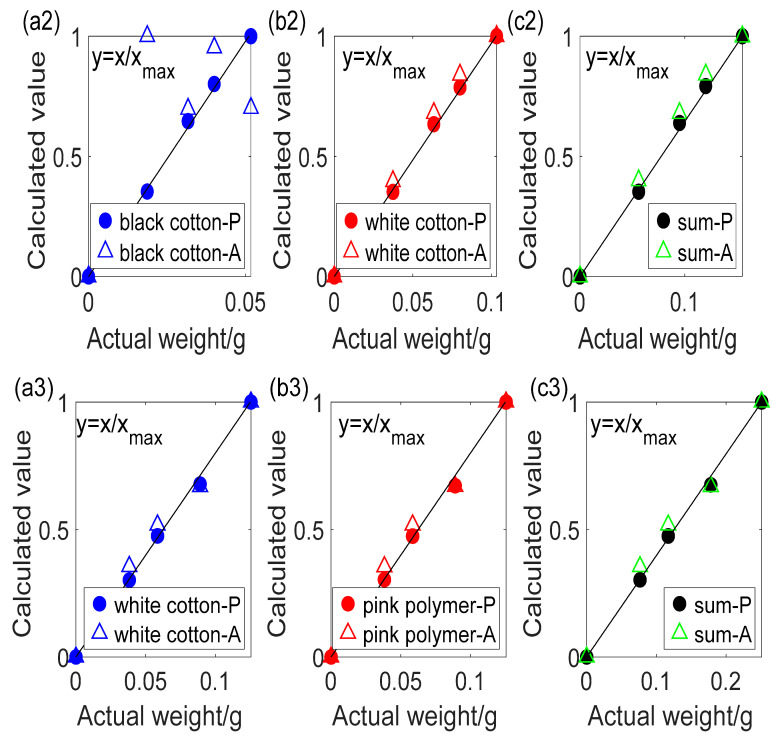
Comparison between actual weight and calculated relative thickness, named calculated value in Figure of each component and their sum for color mixed wool (**a1**–**c1**), cotton (**a2**–**c2**) and cotton and polymer mixed (**a3**–**c3**) fibers.

To make sure the algorithm’s applicability in fiber assemblies, cotton, wool and polymer colored fiber assemblies were used to compare with the common L-B law. Primary fibers, white and black colored cotton, grey and yellow colored wool fibers, and pink colored polymer fibers were piled up parallelly with different weight for optical weight with transmission images. Linear regression method was applied to relationship between scattered photometric or absorbance and their weight in R, G, and B channels, respectively. These linear equations for each primary fibers under same light were summed up to construct the estimation equations system, as shown in Figure 2. Relative quantity of each primary fiber could be obtained in gray-yellow blended wool and black-white cotton with ratio of 1:1 and 1:2 respectively as procedure above compared with actual weights from a balance (accurate to 0.001 g), as described in Figure 6. The mean and maximum relative weight deviation ratios of scattered photometric and absorbance are 3.57%, 15.29% and 4.44%, 15.04%, for 2-mixed wool fibers, 1.86%, 5.5% and 27.25%, 176.79% for cotton samples, and 1.46%, 5.31% and 6.65%, 16.63% for cotton and polymer mixed fibers, respectively. Hence, this new proposed method is better in fiber assemblies. The large derivation of black cotton using L-B law in Figure 6(a2) may deduced from random errors of fiber assemblies and ignorance of reflectance. Hence, based on this theory, a new method with digital image technology could be invented for primary fiber quantity distribution from blending fiber beards, which is essential data for the fibrogram for primary fiber length measurement.

## 5. Conclusions

In this study, a scattered optical algorithm was proposed for relative quantity distribution of each monochrome component in color mixed material based on derived K-M theory and the color transmission image. The linear regression method and conservation of scattered light were applied to obtain the estimating equation system on a defined optical variable, scattered photometric P, from transmission images of monochrome item with different weight or thickness. The obtained results were relative quantities to avoid ignorance factors of theoretical surface reflectance, random error, and measured derivation of reflectance of infinite layers. A series of experiments were performed with color-mixed specimens with smooth PET, rough PP, PP\PET mixed films, cotton, and wool fiber assemblies. Results show that this algorithm performs better than the commonly used L-B theory, especially in smooth PET\rough PP mixed materials and fiber assemblies. Therefore, this optical algorithm shows a potential application in assessing the primary fiber length of blending fibers, as well as testing the evenness of scattering film and fiber assembly, especially hollow fibers and other fiber materials with shape modifications for functional application [17,18,19], as well as to support the fibrogram of fiber beards for fiber length testing. Based on this theory, a new method with digital image technology could be invented for primary fiber length measurement from blending fiber beards, having the characteristics speed, high accuracy, and low cost.

## Figures and Tables

**Table 1:** Information of Monochrome Films.

Number	Color	Image	Thickness/mm	Material	Surface
1#	Blue	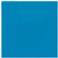	0.02	PET	S
2#	Yellow	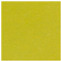	0.02	PET	S
3#	Pink	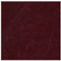	0.35	PET	S
4#	Cyan	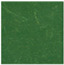	0.35	PET	S
5#	White	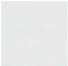	0.01	PET	S
6#	Dark blue	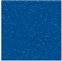	0.5	PP	R
7#	Dark Yellow	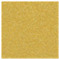	0.5	PP	R

S and R means smooth and rough respectively.

**Table 2:** Information of Mixed Multilayer Films.

		Image	Order	Ratio	Group	Material	Surface
A#	a#	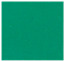	2#1#	1:2	4	PET	S
	b#	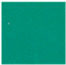	1#2#	3:1	3	PET	S
	c#	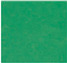		1:1	3	PET	S
	d#	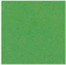		1:2	4	PET	S
B#	e#	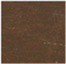	4#3#	1:1	3	PET	S
C#	f#	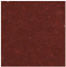	4#5#3#	1:1:1	2	PET	S
D#	g#	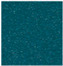	6#7#	1:1	3	PP	R
E#	h#	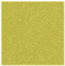	7#4#	1:1	7	PP\PET	R\S
	i#	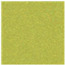		1:2	4	PP\PET	R\S

S and R means smooth and rough respectively.

**Table 3:** *r*_∞_ of Monochrome and Mixed Films under Monochrome Light.

Number	*r*_∞_ of Monochromatic Specimen/%			Number	*r*_∞_ of Mixed Specimen/%		
	R	G	B		R	G	B
1#	2.10	50.85	70.02	a#	27.03	55.84	42.43
2#	74.35	71.79	22.13	b#	26.50	55.89	46.89
3#	22.49	10.12	12.01	c#	35.33	59.82	40.22
4#	29.37	40.58	23.73	d#	44.64	62.54	35.07
5#	85.19	87.98	93.58	e#	30.15	26.93	20.40
6#	15.80	31.11	53.62	f#	32.93	16.85	14.82
7#	76.49	66.95	34.00	g#	17.40	32.83	37.44
				h#	73.47	71.35	33.41

**Table 4:** The relative thickness deviation ratios of each component.

Order	Ratio	Scattered Photometric		Absorbance	
		Mean Deviation Rate%	Max Deviation Rate%	Mean Deviation Rate%	Max Deviation Rate%
2#1#	1:2	1.53	5.09	4.26	20.4
1#2#	3:1	2.56	6.21	4.029	19.57
	1:1	6.81	14.03	12.45	42.24
	1:2	3.36	8.76	6.35	23.48
4#3#	1:1	2.72	6.35	5.12	24.63
4#5#3#	1:1:1	2.28	6.86	1.7	6.74
6#7#	1:1	1.94	6.13	4.08	12.05
7#4#	1:1	2.89	14.38	17.07	78.31
	1:2	4.09	12.01	10.69	42.72

## Data Availability

Not applicable.
